# Bridging the Gap from Enterotypes to Personalized Dietary Recommendations: A Metabolomics Perspective on Microbiome Research

**DOI:** 10.3390/metabo13121182

**Published:** 2023-12-02

**Authors:** Madeline Bartsch, Andreas Hahn, Shoma Berkemeyer

**Affiliations:** 1 NutritionLab, Faculty of Agricultural Sciences and Landscape Architecture, Osnabrueck University of Applied Sciences, Am Kruempel 31, 49090 Osnabrueck, Germany; m.bartsch@hs-osnabrueck.de; 2 Institute of Food Science and Human Nutrition, Leibniz University Hannover, 30167 Hannover, Germany; hahn@nutrition.uni-hannover.de

**Keywords:** metabolomics, enterotypes, personalized dietary recommendations, acetate, propionate, butyrate, tryptophan, bile acids

## Abstract

Advances in high-throughput DNA sequencing have propelled research into the human microbiome and its link to metabolic health. We explore microbiome analysis methods, specifically emphasizing metabolomics, how dietary choices impact the production of microbial metabolites, providing an overview of studies examining the connection between enterotypes and diet, and thus, improvement of personalized dietary recommendations. Acetate, propionate, and butyrate constitute more than 95% of the collective pool of short-chain fatty acids. Conflicting data on acetate’s effects may result from its dynamic signaling, which can vary depending on physiological conditions and metabolic phenotypes. Human studies suggest that propionate has overall anti-obesity effects due to its well-documented chemistry, cellular signaling mechanisms, and various clinical benefits. Butyrate, similar to propionate, has the ability to reduce obesity by stimulating the release of appetite-suppressing hormones and promoting the synthesis of leptin. Tryptophan affects systemic hormone secretion, with indole stimulating the release of GLP-1, which impacts insulin secretion, appetite suppression, and gastric emptying. Bile acids, synthesized from cholesterol in the liver and subsequently modified by gut bacteria, play an essential role in the digestion and absorption of dietary fats and fat-soluble vitamins, but they also interact directly with intestinal microbiota and their metabolites. One study using statistical methods identified primarily two groupings of enterotypes *Bacteroides* and *Ruminococcus*. The *Prevotella*-dominated enterotype, P-type, in humans correlates with vegetarians, high-fiber and carbohydrate-rich diets, and traditional diets. Conversely, individuals who consume diets rich in animal fats and proteins, typical in Western-style diets, often exhibit the Bacteroides-dominated, B-type, enterotype. The P-type showcases efficient hydrolytic enzymes for plant fiber degradation but has limited lipid and protein fermentation capacity. Conversely, the B-type features specialized enzymes tailored for the degradation of animal-derived carbohydrates and proteins, showcasing an enhanced saccharolytic and proteolytic potential. Generally, models excel at predictions but often struggle to fully elucidate why certain substances yield varied responses. These studies provide valuable insights into the potential for personalized dietary recommendations based on enterotypes.

## 1. Introduction

In the past two decades, advances in high-throughput DNA sequencing have propelled research into the human microbiome and its link to metabolic health. Numerous studies emphasize how changes in the gut microbiota composition correlate with metabolic disorders like obesity [1,2], Type 2 Diabetes Mellitus (T2DM) [3,4], and cardiovascular diseases [5,6]. These shifts in the complex microbiome ecosystem result from various factors, including age [7], gender [8], geographical origin [9], genetics [10], and environmental influences, such as gastrointestinal transit time [11,12,13] and medication use [14], with dietary choices assuming a particularly influential role [15]. 

To enhance our understanding of the complex interrelationship between dietary patterns, the gut microbiome, and their impact on host metabolism and health, researchers have introduced the concept of enterotypes as a valuable classification framework [16,17]. Enterotypes categorize individuals based on the compositional attributes of their gut microbiota. However, understanding the functional implications of these enterotypes on host metabolism requires the integration of metabolomics, a systematic discipline concerned with the study of small molecular compounds or metabolites.

Metabolomics furnishes a comprehensive overview of the spectrum of metabolites produced by the gut microbiota in response to diet [15,18,19]. 

In this review, literature research focused on the keywords “diet”, “microbiome analysis”, “metabolomics”, “enterotypes”, “*Prevotella*”, and “*Bacteroides*” in the PubMed database. We examined the literature from 2011 to the present to align with the first paper on enterotypes. It is important to note that the methodological section, exploring microbiome research and connections between diet and microbial metabolites, considers earlier publications to provide a holistic understanding of the topic. 

We delve into microbiome analysis methods, with a specific emphasis on metabolomics. Additionally, we investigate the impact of dietary choices on the production of microbial metabolites and offer an overview of studies examining the connection between enterotypes and diet. Finally, we evaluate how metabolomics can enhance personalized dietary recommendations, drawing insights from the intricate relationship between enterotypes and diet.

## 2. Metabolomic Approaches to Decode Diet–Microbiome Relationships

To understand complex relationships between human nutrition and the composition of the microbiome, amplicon-based sequencing of 16S ribosomal ribonucleic acid (rRNA) has been a widely used approach for analyzing microbiome composition. Significant contributions in this area have been made by the Human Microbiome Project initiative, which played a crucial role in establishing fundamental frameworks for a microbiota analysis [20]. The amplicon-based sequencing involves amplifying hypervariable regions within the 16S gene, unique to specific genera, followed by sequencing. However, it is limited to sequencing bacteria and archaea and provides restricted taxonomic resolution [21]. As this technique only allows conclusions about the presence and relative abundances of particular bacterial genera, it does not reveal insight into their functional potential [22]. 

As the cost of sequencing has decreased, there has been a notable shift towards metagenomic analyses of microbes, exemplified by the impactful MetaHIT project [23]. The metagenomic approach not only characterizes the taxonomic composition of a sample but also reveals its functional metabolic capabilities. For instance, it enables the identification of genes encoding enzymes responsible for the degradation of specific food components [24]. However, metagenomics alone cannot provide information about microbial gene expression activity. To address this limitation, researchers increasingly employ large-scale metagenomics, metaproteomics, and metatranscriptomics to gain deeper insights into how microbial communities respond to changing environmental conditions over time. Metaproteomics enables a detailed investigation of microbial proteins and provides insights into expressed genes and microbial functions. In 2008, Verberkmoes et al. first employed this method, which analyzes the entire set of proteins (proteome) encoded by the microbes in a sample. High-resolution mass spectrometry combined with liquid chromatography enabled the separation and identification of peptide mixtures. By connecting peptide sequences to genomic databases, specific proteins can be linked to the microorganisms responsible for producing them [25]. Although only a portion of the protein material is currently reliably identified, ongoing efforts to standardize and catalog these data enhance the utility of metaproteomics in studies exploring the meta-omics landscape [26]. 

As part of the progression in advanced microbiome analysis techniques, Booijink et al. conducted the initial metatranscriptome analysis of the human fecal microbiome [27]. Metatranscriptomics provides tractable links between the genetic potential of a microbe and its molecular activity [28]. Metatranscriptomic methods involve sequencing RNA actively transcribed by a microbial community, presenting more complex technical challenges than metagenomics, including the maintenance of RNA integrity and the selective isolation of messenger RNA. Despite these obstacles, the significance of metatranscriptomics in microbiome research continues to grow [29]. 

This seamless progression from amplicon-based sequencing through metagenomics to metaproteomics and metatranscriptomics established a comprehensive foundation for exploring the microbiome’s functional aspects. However, to gain a truly holistic understanding of its role in human health, we delve further into the realm of metabolomics.

Metabolomic techniques provide insight into the overall metabolic status and interactions occurring within the microbiome. A pivotal component of the interaction of the gut microbiota with the host physiology is the variety of small molecules (metabolites) produced by the microbial community and the host. Due to the diverse characteristics of metabolites in terms of size, polarity, and abundance, identifying and quantifying the complete pool of metabolites remains a challenge. Metabolomics employs various technologies to measure defined sets of known metabolites (targeted metabolomics) or to perform a comprehensive analysis of the metabolome (untargeted metabolomics). 

Targeted metabolomics offers higher sensitivity and more precise quantification by using internal standards and normalization techniques. Untargeted metabolomics detects a wider range of metabolites, including previously unknown ones, which can lead to novel hypotheses about complex metabolic pathways. Combining both untargeted and targeted metabolomics approaches can provide a comprehensive view of the metabolome. While handling the vast amounts of data generated by these techniques is challenging, metabolomics offers a direct and informative way to understand the physiological state of the host and the interface between the host and its microbiome [30]. Metabolite profiling is increasingly used in research to understand the effects of specific gut microbial changes and the impacts of microbiome-derived or host–microbiota co-substrates on human health. There are several methods to measure microbial metabolites in stool, saliva, urine, blood, or food [19,30].

Within this array of methods, mass spectrometry (MS) became more popular in scientific studies due to its ability to detect metabolites with high sensitivity, impartiality, and efficiency [31]. To accurately identify and quantify metabolites in complex biological samples, MS is often preceded by chromatographic separation to improve sample resolution. Gas chromatography–mass spectrometry (GC-MS) is particularly suitable for volatile metabolites like short-chain fatty acids (SCFAs) but can also handle non-volatile compounds like sugar metabolites, amino acids, and their derivatives when coupled with specific chemical derivatization steps. On the other hand, liquid chromatography–mass spectrometry (LC-MS) is widely used for both non-polar (e.g., bile acids and lipids) and polar (e.g., purines, amino acids, vitamins) metabolite analyses. LC-MS operates at lower temperatures and uses gentler ionization methods than GC-MS, making it suitable for larger, non-volatile, and less stable metabolites [19]. 

Nuclear magnetic resonance (NMR) spectroscopy is another method used in metabolomics, albeit with lower sensitivity compared to mass spectrometry. NMR allows the quantification of abundant metabolites with relatively simple sample preparation and provides structural information, which is valuable for identifying new microbiota-related compounds. However, identifying the structure of spectral hits can be challenging due to the diversity of microbial products, many of which are not well characterized. Both MS and NMR spectroscopy enable an untargeted metabolite analysis and can be used to trace isotopes to investigate nutrient assimilation and metabolic activity in the microbiota. Despite these challenges in characterizing specific metabolic flux within the microbiota using isotopic labeling—a hurdle arising from the shared presence of many metabolites among the host and various microorganisms in the microenvironment [19]—microbiome research has predominantly centered on analyzing metabolites in serum and feces. However, recent literature highlights the significance of also examining the effects of metabolites found in urine [32] and saliva [33] on overall health. Limiting the assessment to a single biofluid may provide researchers with a narrow and potentially misleading perspective. A more comprehensive understanding can be achieved by considering multiple biofluids in these studies [18]. 

Given the complexity and myriad individual factors that influence the interactions between the diet, microbiota, and host, it is critical to bring all these data together to provide the best possible insights and, in the best case, integrate them into public health policy and dietary recommendations. Combining metabolomics with artificial intelligence (AI) and machine learning (ML) techniques will revolutionize our understanding of the microbiome and its importance for personalized medicine, biomarker discovery, drug development, and nutrition. AI and ML algorithms can help researchers integrate data from various sources, such as genomics, metagenomics, and metabolomics, to provide a holistic understanding of microbial communities. This can aid in identifying metabolic pathways, interactions, and functions within the microbiome [34]. Metabolomics datasets are often characterized by many variables, making a data analysis and interpretation challenging. Fortunately, AI techniques offer valuable assistance in this domain by efficiently identifying the most relevant metabolites for specific research questions. By leveraging AI, researchers can streamline feature selection, enhancing their ability to extract meaningful insights from complex metabolomics data. ML models can be harnessed to construct predictive models that establish connections between microbiome composition, metabolite profiles, and various health outcomes or disease states. These models, driven by AI, illuminate the potential roles of the microbiome in numerous physiological processes, shedding light on previously obscured links between microbial communities and human health [35]. 

In the realm of metabolomics research, ethical considerations are paramount. Researchers must uphold fundamental principles to ensure the responsible conduct of both metabolomics methods and AI/ML applications. Privacy and informed consent are foundational. In a metabolomics analysis, researchers must ensure participant privacy and obtain informed consent when collecting biological samples. Participants must understand how their biological samples will be used and the potential implications of the research. In an AI-driven metabolomics analysis, particularly in personalized medicine, individuals should be informed about how AI algorithms will use their data to shape healthcare decisions. Obtaining informed consent is crucial for upholding individuals’ rights and autonomy [36]. Industry influence is an overarching ethical issue across these fields. Disclosure of financial ties and potential conflicts of interest is critical to maintaining transparency and trust in research and healthcare. Adequate regulatory oversight is necessary to ensure that industry-driven research and products meet rigorous standards and prioritize public health over profit. To effectively address these ethical concerns, it is crucial to foster collaboration among researchers, ethicists, policy makers, and healthcare professionals. Together, they can develop guidelines, regulations, and best practices that promote the responsible and equitable advancement of metabolomics, microbial research, and personalized nutrition, maximizing their benefits for society while minimizing potential risks [37].

## 3. The Tight Interaction between Diet, the Gut Microbiome, and Its Metabolites

Nutrients significantly impact microorganisms in the gut, either aiding or hindering their growth. Moreover, certain gut microbes extract energy from specific dietary elements, giving them a competitive edge. This complex interaction between diet and the gut microbiome also shapes the production of diverse microbial metabolites, subsequently affecting our overall health.

### 3.1. Carbohydrates and Dietary Fiber

Indigestible carbohydrates, obtained from various dietary—mainly plant-based—sources, play a key role in this process [38,39]. These substances, also known as microbiota-accessible carbohydrates (MACs) were defined by Sonnenburg et al. [40]. They include dietary fibers such as resistant starch, inulin-type fructans, fructooligosaccharides, or pectin. They reach the colon without being digested because the human body lacks the necessary enzymes for their degradation. In contrast to humans, the microbiome possesses an array of glycoside hydrolases and polysaccharide lyases, collectively referred to as carbohydrate-active enzymes (CAZymes) [15]. In the degradation of complex dietary carbohydrates, SCFAs are the primary end products, along with the production of CO_2_ and H_2_. SCFAs are arguably the most extensively studied microbial metabolites, with numerous effects on human metabolism [41,42]. The functions of SCFAs in maintaining host well-being and influencing health conditions are extensive. SCFAs govern various physiological and biochemical processes within the body. These include upholding the integrity of the innate gut barrier at the colonic epithelium and mucus levels [43], regulating gut motility [41], and controlling the secretion of important gut hormones like Peptide YY (PYY) [44], serotonin [45], gastric inhibitory peptide [46], and glucagon-like peptide 1 (GLP-1) [47]. Moreover, SCFAs are involved in chromatin regulation [48,49], intricate gut–brain connections [42], and immune responses [50]. To exert these metabolic effects, binding to G-protein coupled receptors 41 and 43 (GPR41/43), particularly expressed by the enteroendocrine L cells, plays a crucial role [41]. Acetate, propionate, and butyrate constitute more than 95% of the collective pool of SCFAs. They are found in the intestinal tracts of humans at a proportionate molar ratio of approximately 60:20:20. Outside the colon, this ratio changes to 180:5:1, indicating that most propionate and butyrate are used where they are produced. However, this ratio shifts in individuals consuming Western diets high in fat and low in fiber, resulting in lower peripheral acetate levels [51]. This change is significant due to acetate’s role in metabolic diseases, particularly T2DM. Acetate is synthesized by most enteric bacteria through the pyruvate-to-acetyl-CoA pathway and by bacteria such as *Blautia hydrogenotrophica*, *Clostridium*, and *Streptococcus* spp. through the Wood–Ljungdahl pathway [52,53,54]. The health effects of acetate are debated. Some studies link it to reduced appetite and weight loss via GPR41/43 interaction [55] and enhanced insulin sensitivity [56], while others suggest its role in promoting obesity as a substrate for the liver [57] and adipose tissue fat [58] production. Some studies even associate acetate with cancer cell survival under hypoxic conditions [59]. The conflicting data on acetate’s effects may result from its dynamic signaling, which can vary depending on physiological conditions and metabolic phenotypes [51,60]. Propionate is produced by *Bacteroides* spp., *Phascolarctobacterium succinatutens*, *Dialister* spp., and *Veillonella* spp. through the succinate pathway and via the acrylate pathway by *Megasphaera elsdenii*, *Coprococcus catus*, *Salmonella* spp., *Roseburia inulinivorans*, and *Ruminococcus obeum* [52,53,54]. Colonocytes use propionate for intestinal gluconeogenesis via the free fatty acid receptor 3 (FFAR3) signaling pathway, or is absorbed into the portal system and taken to the liver for hepatic gluconeogenesis [61]. Human studies suggest that propionate has overall anti-obesity effects, as it can increase post-prandial GLP-1 and PYY levels, reduce weight gain, intra-abdominal fat, and intrahepatocellular lipid content, and prevent insulin sensitivity issues [61]. Propionate also exhibits anti-inflammatory properties by reducing the release of interleukin-8 (IL-8) and tumor necrosis factor α (TNF-α) from neutrophils [62]. Of all the SCFAs produced in the gastrointestinal tract by fermentation, butyrate is particularly noteworthy. It has garnered extensive attention in scientific research due to its well-documented chemistry, cellular signaling mechanisms, and various clinical benefits [51]. Butyrate can be converted via the condensation of two molecules of acetyl-CoA and subsequent reduction to butyryl-CoA via the classical pathway by phosphotransbutyrylase and butyrate kinase. Butyryl-CoA can also be converted to butyrate via the butyryl-CoA/acetate-CoA transferase pathway. Important butyrate-producing genera and species are *Coproccocus genus*, *Anaerostipes* spp., *Eubacterium genus*, *Faecalibacterium prausnitzii*, and *Roseburia* spp. [52,54,63]. Butyrate serves as the primary fuel source for mature colon cells, supporting colon health, and acts as a microbial metabolite with strong anti-inflammatory properties, both locally and systemically [64]. Additionally, butyrate plays a crucial role in regulating local and systemic immunity [65], maintaining mucosal integrity [66], and inhibiting cellular-level neoplastic changes [64]. Butyrate, similar to propionate, has the ability to reduce obesity by stimulating the release of appetite-suppressing hormones and promoting the synthesis of leptin [67]. 

### 3.2. Proteins and Amino Acids 

When fermentable fibers become scarce, microbes adapt by utilizing less favorable energy sources for their growth, such as amino acids from dietary or endogenous proteins or dietary fats [68]. Although most of the protein absorption in humans occurs in the small intestine, about 5–10% of dietary protein is not absorbed through the ileum and enters the colon as proteins and peptides [69,70]. The extent to which the gut microbiota utilize amino acids depends primarily on substrate availability and the luminal milieu. For example, increased pH in the colon [71] and decreased availability of carbohydrates [72] are associated with increased rates of bacterial fermentation of proteins. Among the bacteria exhibiting proteolytic properties are genera such as *Bacteroides*, *Clostridium perfringens*, *Propionibacteria*, *Streptococci*, *Bacilli*, and *Staphylococci* [70,73]. The primary pathway of amino acid fermentation in the colon involves deamination, producing SCFAs and ammonia. The liver converts ammonia into urea, which is excreted in urine. The degradation of proteins by the microbiota results in significantly lower production of SCFAs than that derived from carbohydrates [70]. Approximately 30% of the substrates are converted into short SCFAs and branched-chain fatty acids (BCFAs) such as isobutyrate, 2-methylbutyrate, and isovalerate, as well as intermediates such as lactate and succinate. These are often used as indicators of protein fermentation. There is limited knowledge about the role of BCFAs in humans and metabolic health [41,74]. However, recent research has highlighted the importance of their precursor compounds, branched-chain amino acids (BCAAs), in obesity, insulin resistance, and T2DM. Elevated BCAA levels in the blood are linked to insulin resistance across diverse populations and regions. They tend to co-occur with other metabolites like aromatic amino acids (phenylalanine and tyrosine) and acylcarnitines, all of which indicate an excess of BCAAs in the body through different processes [75,76]. 

While the microbial degradation of the BCAAs valine, leucine, and isoleucine is associated with rather negative effects, recent data suggest that tryptophan metabolites, derived from dietary sources such as meats and nuts, play a significant role in maintaining intestinal health [19]. Tryptophan is one of the nine essential amino acids humans cannot synthesize and must obtain by dietary protein sources. The small amount that does reach the colon is converted into indole, various indole derivates, serotonin, and kynurenine under the direct or indirect control of the microbiota [77]. Tryptophan metabolites exhibit diverse functions, such as antimicrobial properties against various bacteria and parasites [78], modulation of the immune system through the aryl hydrocarbon receptor (AHR) [79], and preservation of intestinal balance by promoting mucous production and goblet cell differentiation [78]. They also affect systemic hormone secretion, with indole stimulating the release of GLP-1, which impacts insulin secretion, appetite suppression, and gastric emptying. Indole propionic acid (IPA) acts as an antioxidant by scavenging free radicals [65]. 

### 3.3. Dietary Fat and Bile Acids

In the past, experts underestimated the influence of dietary fat on the gut microbiota, believing that most fat digestion and absorption occurred in the small intestine and little to no dietary fat reached the colon in healthy individuals. This belief was rooted in the understanding that bacterial populations in the digestive tract increased as one moved from the small intestine to the colon, making significant interaction between dietary fat and gut microbiota seem unlikely [80]. Recent research has challenged the idea that dietary fat does not affect the gut microbiota. Gabert et al. (2011) have shown that a substantial portion (approximately 7%) of dietary fatty acids labeled with carbon-13 are excreted in the stools of healthy individuals. Interestingly, more than 86% of these excreted fatty acids are free fatty acids, indicating that digestion failure is not the cause of fat in stool. Digestive lipases can break down triglycerides into free fatty acids. These findings suggest that dietary fat significantly impacts the gut microbiota, contradicting prior assumptions [81]. 

A diet rich in fat can significantly modify the composition of the gut microbiota, resulting in an overrepresentation of bacteria that express lipopolysaccharides (LPSs) [82]. This results in a pro-inflammatory condition known as metabolic endotoxemia. Metabolic endotoxemia, characterized by elevated levels of LPS in the bloodstream, leads to pro-inflammatory responses in both mice and humans. This inflammatory state is mediated through toll-like receptor 4 (TLR4) and CD14 in hematopoietic cells, culminating in weight gain, increased adiposity [83], elevated inflammatory markers in white adipose tissue (WAT) macrophages [84], and insulin resistance [85]. Simultaneously, metabolic endotoxemia is associated with increased gut permeability, possibly due to reduced expression of genes encoding tight junction proteins [86,87,88]. Intriguingly, these adverse effects appear to be specific to saturated fat consumption. Mice fed a diet rich in lard (saturated fat) exhibit an overabundance of specific bacterial taxa, including *Bacteroides*, *Turicibacter*, and *Bilophila* spp., which can promote WAT inflammation, adiposity, and impaired insulin sensitivity [89]. Mice on an unsaturated-fish-oil-rich diet exhibit an expansion of *Bifidobacterium*, *Akkermansia*, and *Lactobacillus* spp., with no discernible metabolic impairments. The transplantation of these distinct microbial compositions into germ-free mice replicates the respective metabolic phenotypes, underscoring the pivotal role of the gut microbiota in mediating the differential effects of dietary fat types on host health [89]. SCFAs’ beneficial effects in the context of fiber intake were previously explained. Studies describing the relationship between dietary fat and SCFAs provide partly contradictory results. Fava et al. reported humans consuming a saturated-fat-rich diet with higher levels of fecal SCFAs were associated with lower fecal energy content, suggesting that dietary fat can contribute to obesity by increasing energy harvest [90]. However, it is essential to note that this observation lacks direct evidence linking SCFAs to weight gain. A high-fiber diet, which also raises SCFA levels, is associated with reduced weight gain in humans [91]. The relationship between SCFAs, dietary fat, and obesity is multifaceted and not completely understood. Additional research is needed to clarify the precise role of SCFAs in metabolic syndrome and their interaction with dietary fat compared to fiber intake.

To facilitate the absorption and excretion of dietary fats, bile acids (BAs) are needed for solubilization by micelle formation in the small intestine. Not only do bile acids play an essential role in the digestion and absorption of dietary fats and fat-soluble vitamins, but they also interact directly with intestinal microbiota and their metabolites [92]. Bile acids are synthesized from cholesterol in the liver and subsequently modified by gut bacteria. Approximately 95% of bile acids are reabsorbed in the distal ileum and returned to the enterohepatic circulation. However, bacterial deconjugation, dihydroxylation, and dehydrogenation prevent their reabsorption into enterocytes, allowing about 5% of BAs to proceed into the colon. In the colon, these primary BAs (e.g., cholic acid and chenodeoxycholic acid) interact with the gut microbiota, leading to the conversion into secondary bile acids, including deoxycholic acid (DCA) and lithocholic acid (LCA) [93]. Over 50 different BAs have been characterized as products of the interaction between primary BAs, with DCA and LCA being the two most common [94]. Maintaining a balance between primary and secondary bile acids is crucial for host health, as an imbalance can harm the organism. These factors influence the balance, including the host’s microenvironment, antibiotic exposure, diet, and microbiota composition. High-fat diets result in elevated levels of secondary bile acids in the feces and influence how the gut microbiota processes bile acids. This leads to changes in the overall bile acid composition, affecting the activation or inhibition of the bile acid receptor called farnesoid X receptor (FXR) [95]. Recent research suggests that FXR plays a central role in regulating how bile acids influence the development of intestinal tumors, integrating factors such as diet, the microbiome, and genetic predisposition in the risk of hepatocellular carcinoma and colorectal cancer [96]. 

### 3.4. Plant- and Animal-Derived Bioactive Compounds 

Apart from fiber, plants also contain a diverse group of bioactive compounds in our diet, with polyphenols being one of the most significant groups, several of which are linked to various health benefits. These compounds are ubiquitous in dietary sources like fruits, vegetables, grains, tea, coffee, and wine [97,98]. Systematic classification groups polyphenols into distinct phytochemical families based on shared structural features, including phenolic acids, flavonoids, lignans, lignins, coumarins, and stilbenes. The structural complexity of polyphenols is remarkable, with over 9000 distinct flavonoids alone identified, underscoring their structural diversity [99]. Due to their structural complexity, only a limited portion, approximately 10%, of dietary polyphenols are metabolized and absorbed in the small intestine. The remaining 90% continue to the lower gastrointestinal tract, undergoing significant modifications and degradation by the gut microbiota, ultimately enhancing their absorption and bioavailability [100]. Studying polyphenols has been challenging due to the intricacies of their structures and their limited bioavailability. However, evidence suggests that these compounds impact the composition and function of the gut microbial community [99]. Dietary polyphenols exert their effects through various mechanisms, including anti-inflammatory, antioxidant, and antimicrobial properties, and have been associated with positive outcomes in conditions such as cardiovascular disease [101], cancer [102], metabolic disorders [103], Alzheimer’s disease [104], and inflammatory bowel disease [105]. An intriguing hypothesis is that these compounds can have measurable physiological effects despite their low bioavailability, owing to substantial modifications of the parent compounds by the gut microbiota [99]. 

In addition to nutrients primarily present in plant-based foods, nutrients predominantly sourced from animal-derived foods, such as red meat, poultry, fish, and eggs, also interact with the intestinal microbiome. The gut microbiome is vital in metabolizing nutrients like choline, betaine, and l-carnitine into trimethylamine (TMA). TMA is transported to the portal circulation and undergoes subsequent oxidation to form trimethylamine-N-oxide (TMAO) [106]. Li et al. (2022) updated 24 meta-analyses that analyzed the association between circulating TMAO concentration and health outcomes by including 82 additional studies. They identified six associations, including all-cause mortality, cardiovascular disease mortality, major cardiovascular events, hypertension, T2DM, and glomerular filtration rate, which exhibited particularly strong and compelling evidence [107]. The precise mechanism by which TMAO influences this context remains somewhat unclear. In rodent models, dietary TMAO or its precursors have been shown to accelerate arteriosclerosis and platelet aggregation [108]. Conversely, mice specifically fed L-carnitine [109] and choline [110] diets to increase plasma TMAO levels were found to have reduced aortic arteriosclerosis, suggesting potential differences in downstream effects from various nutrient precursors.

## 4. Interindividual Differences in Microbial Responses to Diet According to Enterotypes

Given the complex interplay between a host organism, its resident microbiota, and how it reacts to various dietary elements, it becomes clear that a one-size-fits-all approach to diet is not feasible. The idea of personalized medicine, which recognizes individual variations, should also be implemented when creating tailored dietary plans due to the numerous variables involved [111]. Integrating precision nutrition approaches can decrease variability in outcomes among individuals, a recurrent challenge of nutrition research. Inconsistent results can obscure the assessment of nutritional interventions. Grouping individuals with divergent responses results in an underestimation of the effect magnitude and introduces substantial fluctuations. Precise nutrition aids researchers in comprehending factors contributing to diverse reactions to dietary interventions, facilitating tailored studies for metabolic heterogeneity [112]. Emerging developments in personalized nutrition have unveiled that how an individual’s metabolism reacts to specific foods is distinctly personal and intricately linked to the composition of their gut microbiota. In this context, an efficient approach would involve categorizing individuals based on their gut microbiota composition to enhance our understanding of how different people respond to diets, thus facilitating the navigation of the intricacies of the microbiota. 

In 2011, Arumgam et al. introduced the concept of enterotypes as an approach to stratify microbiota composition. By analyzing 33 qualified samples from diverse populations, including Europeans, Americans, and Japanese people, researchers successfully classified the samples into three distinct and robust clusters, primarily based on the presence and abundance of unique genera. Enterotype 1 featured the dominance of Bacteroides, along with covariant taxa such as *Parabacteroides*, *Alistipes*, and *Bilophila*. Enterotype 2, in contrast, displayed an inverse relationship with *Bacteroides*, featuring a higher prevalence of *Prevotella*, along with covariant bacteria such as *Desulfovibrio* and, occasionally, *Succinivibrio*. Enterotype 3 was primarily associated with *Ruminococcus* and co-occurring taxa, including *Akkermansia* and *Methanobrevibacter*. They extended their analysis of enterotypes to two additional datasets: 85 metagenomes from Danish individuals [113] and 154 pyrosequencing-based 16S sequences from American individuals [114]. Their findings indicated that these datasets also exhibited the characteristic three-cluster division. The third cluster displayed distinct characteristics, mainly driven by related *Clostridiales* groups, including *Blautia* and *unclassified Lachnospiraceae* [16]. In a separate study, Liang et al. analyzed 181 fecal samples from adults in Taiwan, China, revealing three distinct enterotypes within the population. Two of these enterotypes resembled patterns associated with *Bacteroides* and *Prevotella*. However, a third unique enterotype specific to the Asian population emerged, characterized by a prevalence of the *Enterobacteriaceae* family of bacteria. Liang et al. analyzed the diversity of microbial communities using three different clustering methods and produced nine different diversity matrices. These matrices resulted in varying counts of enterotypes based on evaluation criteria [115]. Additional studies support these findings, as they also classified the microbiota into primarily two main enterotypes associated with *Bacteroides* and *Prevotella* [116,117,118]. *Prevotella* and *Bacteroides* are often negatively correlated to each other, suggesting a competition for nutrients in the intestinal ecosystem between these two genera [119,120]. These findings highlight the versatility of the enterotype methodology, which appears to apply to various populations, seemingly independent of factors such as age, gender, cultural background, and geographic location. When analyzing the gut microbiota composition in diverse global populations, a consistent association between Enterotype 1 and Enterotype 2 with other bacterial groups is notably absent [121]. However, regional dietary distinctions likely contribute to subgroups within the enterotypes. This concept gains support from the consistent prevalence of *Bifidobacterium* within the *Bacteroides* enterotype, notably observed in Japan [122]. Following the results presented by Gu et al. [123], the augmentation of *Bifidobacteria* confers advantages to the *Bacteroides* enterotype regarding plasma bile acids and various metabolic parameters, including fasting blood glucose, insulin, and C peptide. Crucially, neither of the enterotypes appears to exhibit a specific vulnerability to disease in general, as both categories have shown equal associations with various health conditions [124,125]. But it is plausible that subgroups within the Bacteroides type may exhibit a lower bacterial load, a factor proposed as a key driver in Crohn’s disease [126]. 

Categorizing enterotypes based on compositional patterns has gained significant prominence in recent years. It offers the promise of simplifying the intricate landscape of the gut microbiome. Moreover, this classification opens new avenues for microbiota-based diagnostics, therapeutic interventions, disease prevention strategies, and personalized dietary recommendations [127]. They are akin to densely populated areas in the complex multidimensional space of microbial communities, and their relevance continues to fuel extensive discourse within the scientific community. Jeffery et al. [128] and Knights et al. [129] highlighted that the microbiome often exhibits continuous gradients of dominant microbial taxa, challenging the idea of rigidly defined enterotypes. Identifying discrete clusters in high-dimensional data is a complex task, requiring robust statistical tests. Variations in dominant genera, like Bacteroides and *Ruminococcus*, often manifest as a continuous spectrum within and between putative enterotypes. Even when distinct effects are observed, as with *Prevotella*, significant variation persists within these suggested clusters [129]. In response to these intricate challenges, Costeas et al. performed an extensive meta-analysis to reconcile conflicting viewpoints regarding enterotypes. Their analysis resulted in a modified concept of enterotypes, offering a more nuanced and adaptable perspective in microbiome research [127]. Koren and colleagues reported that the methodology significantly impacts the categorization of populations into enterotypes, with distance metrics and clustering score methods exerting the most substantial influence. They recommend using at least one absolute scoring method in conjunction with 2–3 different distance metrics to validate the presence of enterotypes. Currently, there is no widely accepted consensus on precisely defining an enterotype. Researchers working with the concept may reach opposing conclusions regarding enterotype presence if they apply different criteria, even when analyzing the same data. To enhance the practical utility of the enterotype concept, standardization in enterotyping methods is necessary, particularly to benefit microbial ecologists and clinicians interested in this field [130]. However, some promising studies suggest that enterotypes may aid in predicting dietary responses.

In 2011, Wu et al. were among the first to explore the connections between dietary variables and enterotypes. They conducted a cross-sectional analysis with 98 healthy volunteers, obtaining 16S rRNA sequencing data from stool samples. They collected dietary information from participants through dietary recalls and evaluated long-term habits using a food frequency questionnaire. Additionally, they conducted a controlled-feeding study involving ten individuals following either a high-fat/low-fiber or a low-fat/high-fiber diet for 10 days. Following the methodology introduced by Arumugam et al., the researchers explored the potential categorization of the study population into distinct clusters. Various statistical methods were employed, with most indicating only two groupings where *Bacteroides* and *Ruminococcus* enterotypes merged. The feeding study demonstrated microbiome composition changes within 24 h, while the enterotype identity remained constant during the intervention [17]. This effect seems to apply not only to short-term studies but also to long-term studies. A 6-month randomized controlled intervention study confirmed this result. They instructed sixty-two obese subjects, aged between 18 and 65, to choose between following the new Nordic diet recommendations or adhering to the average Danish diet, and no alterations in the enterotypes were detectable [119]. Notably, enterotypes strongly correlate with individuals’ long-term dietary patterns. The *Prevotella*-dominated enterotype (P-type) in humans correlates with vegetarians, high-fiber and carbohydrate-rich diets, and traditional diets. Conversely, individuals who consume diets rich in animal fats and proteins, typical in Western-style diets, often exhibit the *Bacteroides*-dominated enterotype (B-type). The functional differences between the two enterotypes emphasize this observation. The P-type showcases efficient hydrolytic enzymes for plant fiber degradation but has limited lipid and protein fermentation capacity. Conversely, the B-type features specialized enzymes tailored for the degradation of animal-derived carbohydrates and proteins, showcasing an enhanced saccharolytic and proteolytic potential [127,131,132]. In vitro studies have also observed functional distinctions, particularly when examining dietary fibers of different chemical structures. Upon exposure to arabinoxylans derived from grain bran, fecal samples associated with the P-type enterotype demonstrate elevated production of short-chain fatty acids, notably propionate, in contrast to the B-type samples [118]. P-type specimens exhibit a diminished capacity for growth when subjected to primary carbohydrate substrates, such as soluble starch, pectin, and xylan, in stark contrast to their B-type counterparts [133]. 

Researchers are increasingly basing their investigations on these findings. A research group from Denmark observed these functional differences in a series of human dietary intervention studies. They report that the *Prevotella* to *Bacteroides* ratio (P/B ratio) is closely related to alterations in body fat [134,135,136] and weight [135,136,137,138,139] (Table 1). A caloric deficit of 500 kcal for 24 weeks led to more significant weight and body fat loss in individuals with a high P/B ratio, while weight loss significantly correlated with fiber intake [135]. Similar observations were made by Zou et al. A 3-week calorie restriction also resulted in a higher BMI loss in P-type individuals [140]. A high-fiber diet, characteristic of traditional dietary patterns in P-type individuals, likely results in more efficient and substantial weight loss [134,136,137,138,139,141]. Kovatcheva-Datchary et al. additionally demonstrated that a barley kernel had a positive impact on glucose and insulin metabolism in high-*Prevotella* individuals. A metagenomic analysis confirmed this by revealing that *Prevotella copri* exhibited an increased potential for fermenting complex polysaccharides after the intervention [142]. These studies focused on the differential response to dietary interventions concerning anthropometric, metabolic, and metagenomic outcomes. Investigations examining the effects of enterotypes on metabolic responses using metabolomics approaches are still uncommon. A large-scale cross-sectional study by Wu et al. (*n* = 1199) used a pipeline to predict metabolic microbial functions. P-type individuals showed elevated metabolic activity involving propanoate, starch, and sucrose, which aligns with findings from in vitro studies. On the other hand, B-type individuals exhibited improved fatty acid metabolism [143]. Kang et al. also employed the pipeline to predict metabolomic microbial functions and examined SCFAs in fecal samples. They also measured incretin hormones—GLP-1 and gastric inhibitory polypeptide (GIP)—and the hunger hormone ghrelin in twelve non-obese adults who followed a low- and high-capsaicin diet for 6 weeks. The response to the intervention varied depending on the enterotype. The intervention increased GLP-1 and GIP concentrations while decreasing ghrelin concentrations in P-type individuals. Higher GLP-1 and GIP concentrations are associated with increased satiety and improved insulin and glucose metabolism. Reduced ghrelin levels are also associated with decreased hunger and increased satiety. In B-types, the intervention resulted in higher *Faecalibacterium* abundances and higher butyrate concentrations [110]. In this case, a metagenomic study providing information on the pathways of both enterotypes and an additional examination of SCFAs in plasma for more insights into systemic effects would be helpful. Metabolomic profiling of serum and urine samples was applied in a study by Shin et al., comparing three different dietary forms in a 4-week cross-intervention study. In P-type individuals, isoleucine levels decreased after traditional nutrition, suggesting an altered BCAA metabolism. After the recommended American diet, acetate concentration in P-types increased, which might result from sufficient fiber intake. Serum carnitine levels increased significantly only in B-type individuals, which suggests an adapted metabolism for animal-based foods. An elevated concentration of urinary dimethylamine, likely resulting from the breakdown of carnitine, further supported this observation [144]. Hur et al. conducted an untargeted and targeted metabolomic analysis of serum samples. The traditional diet led to a reduction in body weight in both P-type and B-type. However, this decrease in body weight was associated with a loss of muscle mass in P-type but not in B-type. Furthermore, the traditional diet led to reduced serum lipid and amino acid concentrations in comparison to the control diet in both enterotypes. The decline in these concentrations was more significant in the P-type than in the B-type [141].

Overall, these studies indicate that there are already promising insights into personalized dietary recommendations based on the composition of the gut microbiome. It seems that P-type individuals’ metabolism may respond more favorably to a fiber-rich diet. Nonetheless, only a few studies support this hypothesis using a metabolomics approach, for example, by measuring SCFAs in various tissues. These include compounds such as TMAO and the metabolic byproducts of amino acid and fatty acid fermentation, and the metabolism of secondary bile acids. Identifying metabolites using metabolomics methods also has its challenges, as previously discussed. Categorizing individuals into enterotypes is a noteworthy simplification of the intricate microbiome complexity, presenting unique technical challenges. In addition to the genera *Prevotella* and *Bacteroides*, the functions and distribution of other bacterial species, along with factors such as transit time and microbial diversity, are pivotal in influencing both health outcomes and the production of microbial metabolites. Furthermore, it is essential to acknowledge that classifying based on a particular genus may not encompass the functions of various strains and clades within bacterial genera, which can exhibit substantial variations and remain unaccounted for in the context of enterotype classification [124]. Creating personalized diet recommendations informed by the microbiome presents a formidable challenge. Human studies encounter complexities stemming from substantial individual variations, the constrained ability to manipulate microbiome composition, and the practical difficulties associated with adhering to experimental dietary regimens. To address these challenges, studies on human nutrition require large participant cohorts, and some metabolic changes necessitate long-duration experiments, which can often be unrealistic. Researchers are addressing this intricate challenge by increasingly relying on AI and machine learning tools. These advanced tools scrutinize data concerning food composition, the microbiome, and human physiological responses, employing this information to forecast the collective impact of these elements on particular outcomes. These models excel at predictions but often struggle to fully elucidate why certain substances yield varied responses. However, when researchers train these algorithms with suitable data, the algorithms can identify essential factors and make accurate predictions about metabolic responses. 

## 5. Conclusions

In conclusion, these studies provide valuable insights into the potential for personalized dietary recommendations based on enterotypes and metabolomics methods. Despite the challenges associated with comprehending the intricacies of the microbiome and its multifaceted functions, the application of metabolomics offers a promising avenue for deciphering these complexities. These findings pave the way for more tailored dietary guidance, offering the potential for improved metabolic responses based on an individual’s unique microbiome composition.

## Figures and Tables

**Table 1:** Characteristics of studies exploring enterotypes and dietary associations.

Author	Nutritional Intervention	Duration	Study Design	Method	Participants	Results
Wu et al. (2011) [17]			Cross-sectional	16S rRNA sequencing	Healthy volunteers (*n* = 98)	B-type: associated with protein and animal fat P-type: associated with carbohydrates
Wu et al. (2011) [17]	High-fat/low-fiber diet Low-fat/high-fiber diet	10 days	Randomized controlled dietary intervention	16S rRNA sequencing and shotgun metagenomics	Healthy volunteers (*n* = 10)	Microbiome composition changed within 24 h Enterotype remained stable
Roager et al. (2014) [119]	New Nordic diet high in fiber or average Danish diet	26 weeks	Post hoc analysis of a randomized controlled dietary intervention	16S rRNA sequencing	Participants with increased waist circumference (*n* = 62)	No change in enterotypes or selected bacterial taxa High P/B group had higher total plasma cholesterol concentrations (*p* < 0.05)
Hjorth et al. (2018) [134]	New Nordic diet high in fiber or average Danish diet	26 weeks	Post hoc analysis of a randomized controlled dietary intervention	16S rRNA sequencing	Participants with increased waist circumference (*n* = 62)	High P/B group had greater body fat loss under new Nordic diet (*p* < 0.001)
Kovatcheva-Datchary et al. (2015) [142]	White wheat flour bread or barley-kernel-based bread	3 days each	Randomized cross-over dietary intervention	16S rRNA sequencing and shotgun metagenomics	Healthy volunteers (*n* = 39)	High P/B ratio benefits response to barley kernels (*p* < 0.05) *Prevotella copri* had increased potential of fermenting complex polysaccharides after barley-kernel intervention (*p* < 0.05)
Hjorth et al. (2019) [135]	500 kcal/d energy deficit diet	24 weeks	Randomized controlled dietary intervention	16S rRNA sequencing	Overweight participants (*n* = 52)	High P/B ratio had greater weight (*p* < 0.001) and body fat (*p =* 0.005) loss High correlation between fiber intake and weight change in high P/B ratio (*p* < 0.001)
Zou et al. (2020) [140]	Calorie restriction (60% of recommended daily intake)	3 weeks	Uncontrolled dietary intervention	**Targeted metabolic profiling** and shotgun metagenomics	Non-obese, healthy adults (*n* = 41)	P-type: higher BMI loss (*p* < 0.05)
Kang et al. (2016) [116]	Low-capsaicin and high-capsaicin diet intervention	6 weeks	Controlled dietary cross-over intervention	16S rRNA sequencing, predicted metabolic activities and **SCFAs in fecal samples**	Non-obese, healthy adults (*n* = 12)	P-type: intervention led to increased plasma GLP-1 and gastric inhibitory polypeptide and decreased ghrelin concentrations (*p* < 0.05) B-type: intervention led to higher *Faecalibacterium* abundance and butyrate concentration (*p* < 0.05)
Wu et al. (2021) [143]			Cross-sectional	16S rRNA sequencing, predicted metabolic activities	Adults with and without metabolic syndrome (*n* = 1199)	P-type: linked to rice-based diet and higher metabolism of propanoate, starch, and sucrose (*p* < 0.05) B-type: linked to Western-style diet and enhanced fatty acid metabolism (*p* < 0.05) Both enterotypes associated with higher lipopolysaccharide biosynthesis activity (*p* < 0.05)
Shin et al. (2019) [144]	Typical Korean diet (TKD), typical American diet (TAD), and recommended American diet (RAD)	Each diet for 4 weeks	Randomized cross-over intervention	16S rRNA and **metabolome profiling of serum and urine samples**	Healthy, overweight adults (*n* = 54)	P-type: TKD decreased serum isoleucine, RAD increased serum acetate (*p* < 0.05) B-type: TAD increased serum carnitine, TAD decreased urinary dimethylamine
De Moraes et al. (2017) [145]			Cross-sectional	16S rRNA sequencing and shotgun metagenomics	Adults with BMI < 40 kg/m^2^ (*n* = 268)	P-type: higher amount of vegetarians (*p* = 0.04) and lower LDL-c concentration (*p* = 0.04) and bacteria, including *Eubacterium*, *Akkermansia*, *Roseburia*, and *Faecalibacterium*, linked to improved cardiometabolic profiles (involving BMI, HDL-c, 2 h glucose, waist, and insulin levels) (*p* < 0.05)
Christensen et al. (2020) [146]	Wheat-bran extract rich in arabinoxylan oligosaccharides (AXOSs) and PUFA from fish oil capsules	4 weeks	Post hoc analysis of a randomized cross-over dietary intervention	16S rRNA sequencing and shotgun metagenomics	Overweight adults with at least one criterion for metabolic syndrome (*n* = 29)	Low P/B group gained weight after AXOS consumption (*p* = 0.009) *Bacteroides cellulosilyticus* abundance predicted weight gain with better precision than P/B ratio (*FDR p* = 0.07)
Hjorth et al. (2020) [137]	New Nordic diet high in fiber or average Danish diet	26 weeks	Post hoc analysis of a randomized controlled dietary intervention	16S rRNA sequencing	Participants with increased waist circumference (*n* = 62)	Combination of low salivary amylase gene copy number and baseline P/B ratio promising predictor for weight loss under fiber-rich diet
Christensen et. al. (2019) [138]	Whole-grain (33 g/d fiber) or refined-wheat diet (23 g/d fiber)	6 weeks	Post hoc analysis of a randomized parallel dietary intervention	16S rRNA sequencing and **SCFAs in fecal samples**	Healthy, overweight adults (*n* = 46)	P-type lost more weight on the whole-grain diet (*p* = 0.013)
Chung et al. (2020) [147]	Habitual diet with AXOS or maltodextrin supplement (15 g/d)	Each for 10 days	Controlled cross-over dietary intervention	16S rRNA sequencing and **SCFAs in fecal samples**	Volunteers ≥ 60 years with normal or slightly obese BMI (*n* = 21)	Inverse proportional *P/B* abundance (*p* = 0.001) P-type: higher mean fiber intake (*p* = 0.03) No differences in calprotectin concentrations, glucose, cholesterol, or triglyceride levels between enterotypes
Christensen et al. (2022) [136]	Whole-grain or refined-wheat diet	6–8 weeks	Post hoc analysis of two randomized controlled dietary interventions	One by 16S rRNA sequencing, one by shotgun metagenomics	Healthy, overweight adults (*n* = 70)	Baseline *Prevotella* abundance predicts body fat change in low-amylase-gene-copy-number group (*p* < 0.05)
Hur et al. (2022) [141]	Korean traditional balanced diet and Western-style diet	Each for 1 month	Randomized cross-over study	16S rRNA sequencing and **untargeted and targeted metabolomic analysis of serum samples**	Healthy, obese women (*n* = 52)	P-type: Western diet associated with higher muscle mass and L-homocysteine, glutamate, and leucine concentrations, traditional diet led to higher hydroxybutyric acid (*p* < 0.05) B-type: Western diet associated with higher serum tryptophan and total cholesterol concentrations, traditional diet was positively associated with glutathione and 3-hydroxybutyric acid concentrations (*p* < 0.05) Traditional diet had greater efficacy in P-type individuals

In this table, words in bold highlight studies that used a metabolomics approach.
